# Promoting Rational Risk Engagement Through Feedback in a Gambling-Analog Learning Environment: A Pilot Study

**DOI:** 10.3390/ijerph23030299

**Published:** 2026-02-28

**Authors:** Yu Cong, Ziping Wang

**Affiliations:** Earl G. Graves School of Business and Management, Morgan State University, Baltimore, MD 21251, USA; ziping.wang@morgan.edu

**Keywords:** youth gambling prevention, decision-making, rational risk engagement, simulation-based learning, calibration of risk sense, immediate feedback

## Abstract

**Highlights:**

**Public health relevance—How does this work relate to a public health issue?**

- Youth exposure to gambling and gambling-like digital environments is increasing, raising concerns about early development of maladaptive risk-taking behaviors.
- This study addresses gambling prevention by examining how feedback influences decision-making under uncertainty in a controlled, educational setting.

**Public health significance—Why is this work of significance to public health?**

- The findings demonstrate that immediate feedback can promote more rational and consistent risk engagement without increasing cognitive burden or suppressing autonomy.
- By targeting decision–calibration rather than risk avoidance, the study offers a preventative approach that complements existing treatment-focused gambling interventions.

**Public health implications—What are the key implications or messages for practitioners, policy makers and/or researchers in public health?**

- Simulation-based, feedback-driven learning environments may serve as scalable tools for youth gambling prevention and risk education.
- Public health researchers and educators can incorporate decision calibration frameworks to design interventions that address risk perception before harmful gambling behaviors emerge.

**Abstract:**

The expansion of legalized gambling and gambling-like digital environments has increased youth exposure to uncertainty, underscoring the need for preventative approaches that promote rational risk decision-making. This pilot study examines whether immediate feedback embedded in a repeated, gambling-analog simulation fosters calibrated participation and improved performance under uncertainty among undergraduate students. Participants from two class sections were assigned by class section to an informed condition with immediate feedback or an uninformed condition without feedback. Across repeated rounds, participants made opt-in or opt-out decisions and completed probability-based tasks. Participants receiving feedback exhibited reduced variability in participation decisions, reflecting more consistent engagement with risk. The informed group also achieved higher accuracy and overall performance on tasks they chose to attempt. These improvements occurred without increases in decision or problem-solving time, suggesting enhanced judgment calibration rather than greater deliberation effort. As a pilot investigation, the findings provide preliminary evidence that feedback-driven simulations may support rational risk engagement in educational settings and warrant further study with larger samples and longitudinal designs.

## 1. Introduction

In recent years, jurisdictions such as Maryland have taken legislative action affecting esports (electronic sports) and related competitive gaming. For example, the Maryland General Assembly passed the Maryland eSports Act (HB48), which formally defines esports competitions and authorizes prize structures while explicitly restricting betting and wagering in these contexts [1]. At the same time, broader efforts to legalize online gambling—including internet gaming and casino-style games—continue to be debated and advanced in the state legislature—e.g., [2,3]. These developments highlight a regulatory environment in which youth are increasingly exposed to gambling-adjacent systems, even in the absence of direct monetary wagering.

The rapid expansion of legalized gambling and gambling-like digital environments has intensified concerns about youth exposure to risk and uncertainty, particularly as such environments increasingly blur the boundary between entertainment and wagering. Young people are now routinely exposed to probabilistic reward structures, high-variance outcomes, and repeated opt-in decisions through online games, loot-box systems, fantasy sports, and competitive gaming platforms, all of which share structural similarities with gambling activities [4,5].

Research on gambling behavior increasingly emphasizes the role of decision-making under uncertainty rather than simple impulsivity or generalized risk preference [6,7]. Decision-making under uncertainty refers to situations in which individuals must evaluate options and make choices when outcomes are probabilistic, ambiguous, or only partially known [8]. In such contexts, individuals rely on heuristics and experience-based learning to guide decisions across repeated encounters with risk [9].

A growing body of evidence suggests that gambling-related harm is closely linked to how individuals perceive and evaluate risk over time [10,11]. Distorted beliefs about odds, overconfidence in personal control, and difficulty updating expectations in response to outcomes have all been identified as factors associated with problematic gambling behavior [12,13]. Importantly, these cognitive distortions can emerge prior to clinical levels of harm, indicating that gambling-related risk is rooted in decision processes that develop through experience [14].

Most existing gambling interventions focus on treatment rather than prevention, addressing biased beliefs or problematic behaviors after gambling-related harm has already occurred—e.g., [15,16,17]. While effective in clinical and regulatory contexts, these approaches are typically implemented once maladaptive patterns are already established. From a preventative standpoint, it is therefore important to understand how risk-related decision processes are initially formed and how they may be shaped before harmful patterns take hold.

Within the gambling literature, intervention strategies have predominantly been developed to manage or correct behavior once problematic patterns are already evident. Cognitive models emphasize the modification of beliefs about chance, control, and randomness, while regulatory and technology-based approaches deploy structural constraints—such as warning messages, expenditure limits, or self-exclusion systems—to alter behavior during gambling episodes [15,16,17,18,19]. From a theoretical perspective, these approaches implicitly assume that maladaptive behavior reflects localized errors in judgment or self-control that can be corrected at the point of engagement. As a result, they provide limited explanatory leverage for understanding how risk evaluations are initially formed, how they evolve through experience, and how they become stabilized over time.

Behavioral decision theory offers a complementary account by conceptualizing gambling as a form of repeated decision-making under uncertainty. Rather than assuming stable preferences or purely impulsive behavior, this framework emphasizes subjective probability estimation, outcome evaluation, and learning from feedback as central drivers of choice [8,20]. When individuals repeatedly encounter uncertain outcomes, they construct internal representations of task difficulty, expected payoff, and personal competence. If these representations are poorly calibrated—overestimating gains, underestimating losses, or misjudging probabilities—individuals may persist in disadvantageous choices even when objective outcomes provide corrective information [12,13]. From this standpoint, problematic gambling can be understood as a failure of calibration between perceived and actual risk rather than as excessive risk-taking per se.

Developmental psychology literature documents that calibration processes may be particularly fragile during adolescence and early adulthood [21,22]. Risk perception and probabilistic reasoning continue to mature during this period, and younger individuals are more likely to rely on heuristic-based judgments, show heightened sensitivity to reward cues, and discount longer-term consequences when evaluating uncertain outcomes [23,24,25]. These characteristics increase vulnerability to environments that involve repeated exposure to uncertain rewards, especially when feedback is delayed, noisy, or misleading.

Health behavior and educational psychology research provides further insight into how such miscalibration may be addressed, e.g., [26]. A substantial body of work demonstrates that judgment and confidence are malleable and can be aligned more closely with objective performance through structured learning experiences and iterative feedback [27,28]. Feedback serves not only to signal correctness, but also to support belief updating by making discrepancies between expectation and outcome salient. Over repeated trials, this process can reduce reliance on unstable heuristics, dampen overconfidence, and promote more consistent decision strategies in uncertain environments.

Simulation-based learning environments are particularly well suited to supporting this calibration process because they embed feedback within repeated, decision-rich contexts that mirror the structure of real-world gambling decisions while avoiding actual monetary risk [29,30]. By allowing individuals to opt in or out, experience gains and losses, and update beliefs over time, simulations create a controlled setting in which the dynamics of uncertainty, feedback, and learning can be observed directly. Importantly, such environments are consequential but safe: decisions have meaningful performance implications without exposing participants to irreversible financial or psychological harm.

Integrating these perspectives suggests that immediate feedback should influence risk-related behavior through two linked mechanisms. First, feedback should promote more calibrated participation decisions by helping individuals refine their assessments of task difficulty, expected payoff, and personal competence across rounds. As uncertainty is reduced and expectations become better aligned with experience, participation behavior should become more stable and less erratic. Second, feedback should improve decision quality among those who choose to engage by supporting learning from outcomes and adjustment of strategies over time.

Accordingly, the central prediction is not simply that feedback will increase or decrease risk-taking, but that it will promote rationally calibrated participation, reflected in reduced variability and greater consistency in opt-in decisions across repeated choices.

**H1.**  *Immediate feedback will promote more rational participation decisions, reflected in lower variability in risk-taking behavior across rounds.*

Feedback should also improve performance among those who opt in. By seeing whether their risk choices and solutions lead to success or failure, participants should adjust strategies, effort, and expectations across rounds. Therefore:

**H2.**  *Participants who receive immediate feedback will earn higher scores and show higher accuracy on opted-in tasks than participants who do not receive feedback.*

## 2. Methods

This section describes the study design, setting, participants, materials, procedures, and measures in the order in which the study was conducted.

### 2.1. Study Design

The study employed a one-factor, between-subjects experimental design. The independent variable was feedback condition, with two levels: an informed condition that received immediate feedback after each task and an uninformed condition that received no feedback until the end of the simulation. The primary dependent variables were (a) participation decisions (opt-in, opt-out, abstain) and (b) performance outcomes on tasks that participants chose to attempt. Participants were assigned to conditions at the class section level. This approach was adopted to maintain instructional consistency within class sessions and to minimize cross-condition contamination during in-class administration.

### 2.2. Setting and Participants

The study was conducted in the United States at a public, historically Black research university located in Baltimore, Maryland. Participants were undergraduate students enrolled in General Education-level courses and were recruited from two intact class-sections during regular class meetings. A total of 60 undergraduate students participated in the study. Participation was voluntary, and no course credit, attendance credit, or academic penalty was associated with participation or non-participation. No participants were excluded from analysis, as no device errors, missing records, or incomplete sessions occurred. Demographically, participants ranged in age from 18 to 24 years (M ≈ 19.35, SD ≈ 1.33). The sample was 56.7% female and 43.3% male and reflected the institution’s student population, with approximately 92% identifying as African American.

### 2.3. Pre-Simulation Survey and Baseline Comparisons

Prior to participating in the simulation, students completed a brief pre-simulation survey. The survey assessed demographic characteristics (age, gender, race/ethnicity), baseline attitudes toward risk, perceived ability to evaluate odds, willingness to guess under uncertainty, and prior exposure to gaming or gambling-like activities. Pre-simulation survey data were used solely to characterize the sample and to assess baseline equivalence between the two class-sections. No survey variables were used to assign participants to conditions, nor were they included as covariates in the primary analyses. Only students who completed the pre-simulation survey were eligible to participate in the simulation. No participants were excluded due to missing survey data, technical difficulties, or non-compliance.

Because participants were assigned to conditions at the class section level, we examined whether the two groups differed at baseline, as presented in Table 1. Analyses of the pre-experiment survey showed no significant differences between the informed (n = 31) and uninformed (n = 29) groups in age, gender distribution, race/ethnicity, prior gaming behavior, or baseline attitudes toward risk, odds, and decision-making (all *p* > 0.10). These comparisons support the assumption that the two class sections were demographically and psychologically comparable prior to the intervention.

### 2.4. Simulation Tasks and Materials

We developed a prototype online platform that allows participants to engage in multiple rounds of simulations. The platform provides a highly configurable simulation environment with prompt feedback for participants. The simulation consisted of a series of probability-based academic tasks designed to mimic key structural features of gambling decisions. Tasks included multiple-choice and fill-in-the-blank questions, with five items of each type. Items were drawn from content commonly covered in General Education coursework and were selected to represent moderate difficulty based on historical classroom performance.

The scoring structure awarded +10 points for a correct response and −5 points for an incorrect response. This asymmetric payoff structure was intentionally chosen to resemble gambling environments in which potential gains exceed losses in nominal value, while uncertainty determines expected outcomes. Participants were informed of the scoring rules and decision structure prior to beginning the simulation so that opt-in decisions could be made with full knowledge of potential consequences.

### 2.5. Procedure and Measure

The simulation was administered synchronously during regular class sessions. After completing the pre-simulation survey, participants scanned a QR code to access the online simulation platform using an internet-enabled personal device, most commonly a smartphone. One investigator used an administrative dashboard to control the timing and progression of the simulation.

Each question was activated simultaneously for all participants. Participants were given 15 s to decide whether to opt in or opt out of attempting the question. Failure to make a selection within this window was recorded as an abstain and resulted in no point change. Participants who opted in were given up to 150 s to submit an answer. Correct answers earned 10 points, while incorrect answers resulted in a 5-point deduction. In the informed condition, participants received immediate feedback after each question, including the correct answer and the points gained or lost. In the uninformed condition, no feedback was provided until the conclusion of the simulation.

All participant actions—including decisions, response accuracy, and response times—were logged automatically in the system’s backend database. Although participants were seated in a shared classroom environment, no communication or collaboration was observed during the simulation. The platform’s individualized, time-locked interface minimized opportunities for interaction.

### 2.6. Statistical Analysis

All statistical analyses were conducted using SAS on Demand, Release 3.1.0. Baseline equivalence between the informed and uninformed groups was assessed using independent-samples *t*-tests for continuous variables (e.g., age) and chi-square tests for categorical variables (e.g., gender and race). To examine the primary hypotheses, one-way analyses of variance (ANOVA) were conducted with group (informed vs. uninformed) specified as the between-subjects factor. Separate ANOVAs were performed to evaluate differences in participation behavior (opt-in, opt-out, and abstain responses) and performance outcomes (points earned) at both the participant and question levels. All statistical tests were two-tailed with a significance level of α = 0.05. Effect sizes were reported using partial eta squared (η^2^) to facilitate interpretation of the magnitude of group differences.

The primary outcome variables were participation behavior and performance. Participation behavior was operationalized as the frequency of opt-in, opt-out, and abstain decisions across tasks. Performance was measured using total points earned and accuracy on tasks that participants chose to attempt. Secondary exploratory measures included decision time (time to opt in or opt out) and solving time (time from question presentation to answer submission). These measures were used to assess whether feedback effects reflected increased deliberation or improved calibration without additional cognitive burden.

## 3. Results

**Figure 1:**
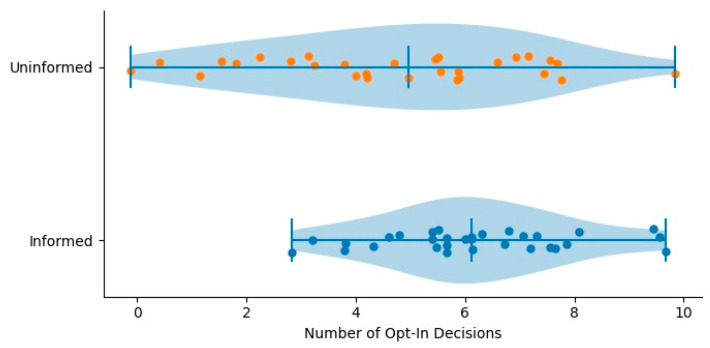
RainCloud Plot of Opt-in Participation (H1).

**Figure 2:**
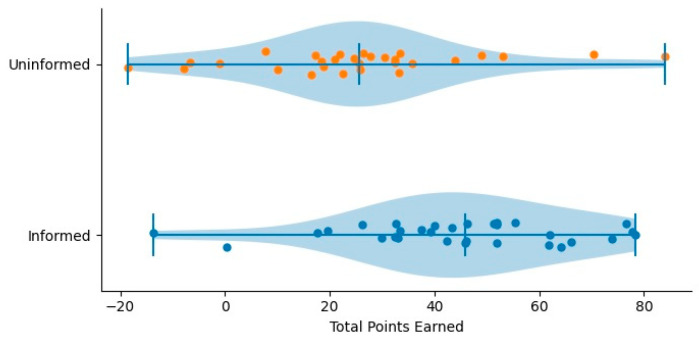
RainCloud Plot of Individual-Level Performance (H2).

The analyses addressed whether immediate feedback influenced (a) participation in risky decisions and (b) performance on tasks that participants chose to attempt. We first examined participation patterns by comparing opt-in, opt-out, and abstain responses across the informed and uninformed groups (Figure 1). We then evaluated performance outcomes by analyzing points earned at both the participant and question levels (Figure 2). Group differences were evaluated using one-way ANOVA with group (informed vs. uninformed) specified as the between-subjects factor.

### 3.1. Participation Behavior

Analyses of participation decisions examined whether immediate feedback produced not only differences in mean opt-in behavior but also more calibrated and consistent participation patterns, as predicted by H1. The results are tabulated in Table 2 and illustrated in Figure 1. Figure 1 presents raincloud plots for opt-in decisions by condition. In addition to higher mean participation in the informed group, the distribution is visibly more compact, illustrating reduced variability in participation behavior relative to the uninformed group.

Specifically, at the question level, the informed group demonstrated substantially lower non-participation (opt-out + abstain) compared to the uninformed group, M = 8.56 (SD = 2.70) versus M = 13.56 (SD = 3.32), respectively. This difference was statistically significant, *p* = 0.0029, partial η^2^ = 0.434, with a 95% confidence interval for the mean difference of [−8.27, −2.73]. Conversely, opt-in behavior was higher among informed participants (M = 22.44, SD = 2.70) than uninformed participants (M = 16.56, SD = 3.54), *p* = 0.0011, partial η^2^ = 0.496, 95% CI [2.47, 9.29].

Although these mean differences indicate that feedback increased willingness to engage with uncertain tasks, the pattern of variability between groups provides additional, theoretically meaningful evidence for H1. The uninformed group displayed substantially greater dispersion in their participation decisions across items, reflecting less stable and less predictable engagement from one task to the next. In contrast, the informed group’s opt-in and opt-out choices were more tightly clustered around a consistent level of participation. This reduced variability is consistent with the prediction that immediate feedback supports calibrated decision-making, enabling participants to align their choices with updated beliefs about task difficulty, potential payoff, and personal competence. Variability patterns are therefore interpreted descriptively and in relation to the study’s theoretical framework, rather than as independent inferential outcomes.

A similar pattern emerged at the participant level. Students in the informed group opted in more frequently (M = 6.58, SD = 1.96) than those in the uninformed group (M = 5.00, SD = 2.61), *p* = 0.0096, partial η^2^ = 0.108, 95% CI [0.40, 2.78]. Again, the uninformed group showed larger between-participant variability, whereas informed participants displayed a narrower distribution of participation frequencies. Taken together, the participation results indicate that the effect of feedback extends beyond simple increases in opt-in rates and is consistent with more stable and calibrated participation behavior across rounds.

### 3.2. Performance Outcomes

Performance outcomes were then examined to test whether the same calibration mechanism that produced more consistent participation behavior also improved the quality of decisions on tasks that participants chose to attempt. The results are tabulated in Table 3 and illustrated in Figure 2. Figure 2 presents raincloud plots for total points earned by condition.

At the participant level, students in the informed condition earned significantly higher total points (M = 44.03, SD = 22.00) than students in the uninformed condition (M = 26.38, SD = 23.37). This difference was statistically significant, *p* = 0.0038, partial η^2^ = 0.135, with a 95% confidence interval for the mean difference ranging from 5.96 to 29.35. This result supports H2 and suggests that feedback aided students in calibrating expectations and adjusting strategies over repeated rounds.

A question-level aggregation produced a similar pattern: questions in the informed condition yielded higher cumulative point totals (M = 136.50, SD = 82.90) than those in the uninformed condition (M = 76.50, SD = 73.86). Although the effect size remained substantial (partial η^2^ = 0.140), this comparison did not reach statistical significance, *p* = 0.1047, likely due to limited statistical power at the question level.

Although feedback produced meaningful gains in mean performance, the standard deviations reveal an important theoretical distinction. Unlike participation behavior—where feedback clearly reduced variability and promoted a more uniform decision strategy—performance outcomes remained widely dispersed, with the informed group showing variability comparable to or greater than the uninformed group (SD = 22.00 vs. SD = 23.37). This divergence indicates that feedback does not homogenize performance. Instead, feedback appears to function as a calibration mechanism, helping students regulate when they take risks, but not fully equalizing how effectively they can solve academic tasks once they choose to engage. In other words, even though students converged toward a more rational and consistent participation pattern, their underlying cognitive abilities, problem-solving strategies, and content knowledge continued to drive substantial differences in accuracy. The enlarged spread among the informed group further suggests a differential benefit: higher-performing students may have leveraged the feedback to refine their strategies more effectively, whereas lower-performing students benefited to a lesser degree. This pattern reinforces the interpretation that feedback improves decision quality but does not eliminate individual differences in competence [23]. Together, the SD profiles across participation and performance provide further support for the interpretation that feedback stabilizes risk-taking behavior while preserving, and perhaps amplifying, the natural heterogeneity of task performance.

### 3.3. Exploratory Analyses

To further probe mechanisms related to calibration (H1) and decision quality (H2), exploratory analyses examined accuracy, decision time, and solving time at the trial level. These analyses were conducted to assess whether feedback effects reflected increased deliberation or improved calibration of judgment under uncertainty.

Trial-level accuracy was significantly higher in the informed condition than in the uninformed condition, *p* = 0.0255. In contrast, no significant differences were observed in decision time (time to opt in or opt out), *p* = 0.257, or in solving time (time from question presentation to answer submission), *p* = 0.359.

Taken together, these results indicate that feedback did not lead participants to slow down or engage in more time-intensive responding. Rather, feedback was associated with higher accuracy and more consistent participation behavior without changes in response time, suggesting improved calibration rather than increased deliberation. This pattern supports the interpretation that feedback sharpened participants’ internal models of task difficulty and expected outcomes, rather than simply inducing more cautious or time-intensive behavior.

These supplemental analyses therefore reinforce the main findings by showing that feedback influenced how decisions were structured and evaluated, not merely how long participants spent making them. In combination with the reduced variability in participation behavior, the accuracy-without-delay pattern provides convergent evidence that immediate feedback promotes a more rational and stable approach to risk in repeated decision contexts.

### 3.4. Summary of Results

Taken together, the results provide consistent support for H1 and H2. Immediate feedback was associated with more stable participation decisions and improved accuracy among participants who chose to engage, without corresponding increases in decision or solving time. These patterns suggest that feedback influenced how participants regulated risk engagement rather than how long or how intensely they deliberated.

## 4. Discussion

This study examined whether immediate feedback embedded in a repeated, gambling-analog simulation can foster more rational engagement with risk among young adults. Drawing on behavioral decision theory and developmental and educational psychology, we conceptualized rational risk behavior not simply as greater or lesser willingness to engage, but as calibrated participation—decisions that are stable, evidence-aligned, and responsive to experience. Across analyses, participants who received immediate feedback exhibited more consistent participation behavior and achieved higher accuracy and overall performance on tasks they chose to attempt, without increases in decision or problem-solving time.

An important feature of the findings is the dissociation between participation behavior and performance outcomes. Immediate feedback was associated with reduced variability in participation decisions, indicating convergence toward a more stable and calibrated approach to risk. At the same time, performance outcomes remained widely dispersed across participants, suggesting that feedback did not homogenize underlying ability or problem-solving competence. This pattern is consistent with prior work in decision science and education showing that feedback often improves judgment calibration and decision strategy without eliminating individual differences in skill or knowledge. In this sense, the present findings suggest that feedback operates primarily as a regulatory mechanism for risk engagement rather than as a general performance equalizer.

These results are particularly relevant in the context of youth gambling prevention. Distorted beliefs about odds, outcomes, and personal control are known to precede problematic gambling behavior, and such distortions often develop before individuals encounter formal gambling environments [31]. The present findings suggest that structured feedback within simulation-based learning environments may help young adults develop a more rational sense of risk by aligning participation decisions with experience-based evidence. Rather than encouraging risk avoidance or indiscriminate engagement, the intervention promoted stability and consistency in decision-making under uncertainty, which may be a critical precursor to healthier gambling-related behaviors.

Importantly, feedback effects were not accompanied by longer decision times or increased deliberation. This suggests that improved outcomes did not arise from greater cognitive effort or caution, but from better calibration of expectations and judgments. Such a mechanism aligns with theories of experiential learning and reinforcement learning, which emphasize the role of timely feedback in shaping internal models of task difficulty and expected value. In applied terms, this finding highlights the potential value of feedback-driven simulations as pedagogical tools that support rational risk engagement without imposing additional cognitive burden.

### Limitations

Several limitations should be considered when interpreting these findings. First, the study was conducted within a single institutional context, and participants were undergraduate students recruited from intact class sections rather than randomly assigned individuals. Although baseline comparisons indicated no meaningful differences between groups, future research should replicate these findings using randomized designs across multiple institutions and populations.

Second, the sample size was modest and reflects the exploratory, pilot nature of the study. While effect sizes were substantial for several outcomes, the study was not powered to detect small effects or to examine more complex interactions. As such, the results should be interpreted as preliminary evidence rather than definitive conclusions about the effectiveness of feedback-based interventions.

Third, the simulation tasks were academic in nature and did not involve real monetary stakes. Although this design choice was intentional to ensure a consequential but safe learning environment, it limits direct generalization to real-world gambling behavior. Gambling that involves real money may introduce additional motivational and emotional factors that influence decision-making under uncertainty. Future studies could extend the present design by incorporating more ecologically realistic gambling scenarios or simulated monetary incentives.

Finally, the study focused on short-term behavioral adaptation within a single session. Whether the observed calibration effects persist over time, transfer to other domains of risk-taking, or influence real-world gambling behavior remains an open question. Longitudinal studies will be necessary to assess the durability and practical impact of feedback-driven simulation interventions.

## 5. Conclusions

Despite these limitations, the present pilot study provides evidence that immediate feedback embedded in a gambling-analog simulation can promote more rational and calibrated engagement with risk among young adults. By stabilizing participation decisions and improving accuracy without increasing cognitive effort, feedback appears to support judgment calibration rather than simple risk aversion or performance enhancement.

These findings contribute to the growing literature on preventative approaches to gambling-related harm by highlighting the potential of simulation-based, feedback-driven learning environments. Such interventions may offer a scalable and educationally grounded pathway for shaping risk perception and decision-making before harmful gambling behaviors emerge. Future research should build on this pilot work by testing larger and more diverse samples, incorporating longitudinal designs, and examining whether calibration gains translate to real-world gambling contexts.

## Figures and Tables

**Table 1:** Baseline Comparisons Between Groups (Pre-Experiment Survey).

Variable	Informed (n = 31)	Uninformed (n = 29)	Sig. of Dif.
Age (years)	19.19 (SD 1.21)	19.52 (SD 1.46)	*p* = 0.345
Gender (% female)	67.70%	44.80%	*p* = 0.150
Race (% African American)	93.50%	89.70%	*p* = 0.335
Plays online games (%)	83.90%	82.80%	*p* = 0.65
Risk attitude	71.00%	72.40%	*p* = 0.60
Willingness to guess (+10/−5)	64%	62%	*p* = 0.65

**Table 2:** Participation Outcomes by Condition.

Outcome	Informed M (SD)	Uninformed M (SD)	*p*	Partial η^2^	95% CI for Mean Difference
Non-participation (question-level)	8.56 (2.70)	13.56 (3.32)	0.0029	0.434	[−8.27, −2.73]
Opt-in (question-level)	22.44 (2.70)	16.56 (3.54)	0.0011	0.496	[2.47, 9.29]
Opt-in (participant-level)	6.58 (1.96)	5.00 (2.61)	0.0096	0.108	[0.40, 2.78]

**Table 3:** Performance Outcomes by Condition.

Outcome	Informed M (SD)	Uninformed M (SD)	*p*	Partial η^2^	95% CI for Mean Difference
Total points (participant-level)	44.03 (22.00)	26.38 (23.37)	0.0038	0.135	[5.96, 29.35]
Total points (question-level)	136.50 (82.90)	76.50 (73.86)	0.1047	0.14	[−14.39, 133.89]

## Data Availability

The datasets presented in this article are not readily available because the data are part of an ongoing study.

## References

[1] Maryland General Assembly *House Bill 48: eSports Act*; Annapolis, MD, USA, 2019. https://mgaleg.maryland.gov/mgawebsite/Legislation/Details/HB0048?ys=2019RS.

[2] Maryland General Assembly *House Bill 1319: Internet Gaming*; Annapolis, MD, USA, 2024. https://mgaleg.maryland.gov/mgawebsite/Legislation/Details/HB1319?ys=2024RS.

[3] CBS News Maryland Lawmakers Renew Push to Legalize Internet Gambling 2025. https://www.cbsnews.com/baltimore/news/online-gambling-maryland-legislation-hb1319/?intcid=CNM-00-10abd1h.

[4] Derevensky J.L., Gupta R., Winters K. (2019). Prevention of youth gambling problems: Evidence-based strategies. J. Gambl. Stud..

[5] Friend K.B., Ladd G.T. (2009). Youth gambling advertising: A review of the lessons learned from tobacco control. Drugs Educ. Prev. Policy.

[6] Brevers D., Cleeremans A., Goudriaan A.E., Bechara A., Kornreich C., Verbanck P., Noël X. (2012). Decision making under ambiguity but not under risk is related to problem gambling severity. Psychiatry Res..

[7] Hoven M., Hirmas A., Engelmann J., Van Holst R.J. (2023). Confidence and risky decision-making in gambling disorder. J. Behav. Addict..

[8] Kahneman D., Tversky A. (1979). Prospect theory: An analysis of decision under risk. Econometrica.

[9] Tversky A., Kahneman D. (1992). Advances in prospect theory: Cumulative representation of uncertainty. J. Risk Uncertain..

[10] Reith G., Dobbie F. (2013). Gambling careers: A longitudinal, qualitative study of gambling behaviour. Addict. Res. Theory.

[11] Goodie A.S. (2005). The role of perceived control and overconfidence in pathological gambling. J. Gambl. Stud..

[12] Clark L. (2017). Disordered gambling: The evolving concept of behavioral addiction. Ann. N. Y. Acad. Sci..

[13] Ciccarelli M., Griffiths M.D., Nigro G., Cosenza M. (2017). Decision making, cognitive distortions and emotional distress: A comparison between pathological gamblers and healthy controls. J. Behav. Ther. Exp. Psychiatry.

[14] Krébesz R., Ötvös D.K., Fekete Z. (2023). Non-problem gamblers show the same cognitive distortions while playing slot machines as problem gamblers, with no loss of control and reduced reality control, though–An experimental study on gambling. Front. Psychol..

[15] Forsström D., Jansson-Fröjmark M., Hesser H., Carlbring P. (2017). Experiences of Playscan: Interviews with users of a responsible gambling tool. Internet Interv..

[16] Newall P.W.S. (2019). Dark nudges in gambling. Addict. Res. Theory.

[17] Newall P.W.S., Weiss-Cohen L., Singmann H., Walasek L., Ludvig E.A. (2022). Impact of the “when the fun stops, stop” gambling message on online gambling behaviour: A randomised experimental study. Lancet Public Health.

[18] Blaszczynski A., Nower L. (2002). A pathways model of problem and pathological gambling. Addiction.

[19] Fortune E.E., Goodie A.S. (2012). Cognitive distortions as a component and treatment focus of pathological gambling: A review. Psychol. Addict. Behav..

[20] Sitkin S.B., Pablo A.L. (1992). Reconceptualizing the determinants of risk behavior. Acad. Manag. Rev..

[21] Weil L.G., Fleming S.M., Dumontheil I., Kilford E.J., Weil R.S., Rees G., Dolan R.J., Blakemore S.J. (2013). The development of metacognitive ability in adolescence. Conscious. Cogn..

[22] Defoe I.N., Dubas J.S., Figner B., Van Aken M.A. (2015). A meta-analysis on age differences in risky decision making: Adolescents versus children and adults. Psychol. Bull..

[23] Furby L., Beyth-Marom R. (1992). Risk taking in adolescence: A decision-making perspective. Dev. Rev..

[24] Reyna V.F., Farley F. (2006). Risk and rationality in adolescent decision making: Implications for theory, practice, and policy. Psychol. Sci. Public Interest..

[25] Gardner M., Steinberg L. (2005). Peer influence on risk taking, risk preference, and risky decision making in adolescence and adulthood. Dev. Psychol..

[26] Williams R.J., Connolly D. (2006). Does learning about the mathematics of gambling change gambling behavior?. Psychol. Addict. Behav..

[27] Brewer N.T., Weinstein N.D., Cuite C.L., Herrington J.E. (2004). Risk perceptions and their relation to risk behavior. Ann. Behav. Med..

[28] Brewer N.T., Chapman G.B., Gibbons F.X., Gerrard M., McCaul K.D., Weinstein N.D. (2007). Meta-analysis of the relationship between risk perception and health behavior. Health Psychol..

[29] Andrà C., Parolini L., Verani M. (2015). Gambling as a context for teaching probability: A teaching experiment with a gambling simulation. Int. J. Math. Educ. Sci. Technol..

[30] Abel J., Cole S., Zia B. (2021). Can experiential learning reduce behavioral biases? Experimental evidence from financial education. J. Dev. Econ..

[31] Yakovenko I., Hodgins D.C., el-Guebaly N., Casey D.M., Currie S.R., Smith G.J., Williams R.J., Schopflocher D.P. (2016). Cognitive distortions predict future gambling involvement. Int. Gambl. Stud..

